# Distributed Data Integrity Verification Scheme in Multi-Cloud Environment

**DOI:** 10.3390/s23031623

**Published:** 2023-02-02

**Authors:** Elizabeth Nathania Witanto, Brian Stanley, Sang-Gon Lee

**Affiliations:** College of Software Convergence, Dongseo University, Busan 47011, Republic of Korea

**Keywords:** blockchain, cloud computing, data integrity, data verification, multi-cloud

## Abstract

Most existing data integrity auditing protocols in cloud storage rely on proof of probabilistic data possession. Consequently, the sampling rate of data integrity verification is low to prevent expensive costs to the auditor. However, in the case of a multi-cloud environment, the amount of stored data will be huge. As a result, a higher sampling rate is needed. It will also have an increased cost for the auditor as a consequence. Therefore, this paper proposes a blockchain-based distributed data integrity verification protocol in multi-cloud environments that enables data verification using multi-verifiers. The proposed scheme aims to increase the sampling rate of data verification without increasing the costs significantly. The performance analysis shows that this protocol achieved a lower time consumption required for verification tasks using multi-verifiers than a single verifier. Furthermore, utilizing multi-verifiers also decreases each verifier’s computation and communication costs.

## 1. Introduction

Cloud computing has led to an ever-growing demand for computing infrastructure, storage, data services, and applications. Examples of famous Cloud Service Providers (CSPs) are AWS, Azure, Google Cloud, and IBM. The National Institute of Standards and Technology (NIST) defines cloud computing as a “model for granting users access to a shared pool of configurable computational resources including networks, servers, storage, applications, and services that can be rapidly provisioned (adapted for new services) with little management or service provider interaction” [1]. Thus, this technology allows computational resources to be employed significantly more efficiently, resulting in faster services being provided.

Over time, there is a problem that arises with the use of a single cloud: a vendor lock-in. Vendor lock-in is a condition where the users become dependent on a single provider [2]. For instance, if a company were to host its resources to a provider and that provider were to go bankrupt, the company might lose all of its data on that cloud. One of the solutions to this problem is utilizing a multi-cloud. The multi-cloud can consist of several private and/or public clouds. Correspondingly, it could enhance data availability.

However, this technology introduces new security challenges, such as unreliable Cloud Service Providers (CSPs) [3,4]. When users outsource their data to CSPs, they provide CSPs with the right to perform any operation on their data. Thus, the user cannot fully control what happens to their data. Moreover, users may already remove the original data from their local storage because it is already stored in CSPs. A major problem with loss of data possession is that CSPs can hide mistakes they made from users for their benefit [5,6]. Besides, CSPs may face internal or external security problems such as accidentally corrupting or eliminating rarely accessed users’ data to reduce storage burden and claims that all data are still stored within the cloud and internal software bugs [3]. In another case, data integrity is at risk if a bad actor successfully compromises the CSPs’ systems [3,4,5,6,7].

Some real cases of data corruption were reported to affect various companies, such as the Amazon S3 breakdown [8], Gmail mass deletion [9], and Amazon EC2 services outage [10,11]. Furthermore, the Privacy Rights Clearinghouse (PRC) reports more than 535 data breaches to several cloud-based email service providers. For instance, these have affected Sony Online Entertainment, Sony PlayStation Network, and Sony Pictures [12]. Lastly, 3.3 million patients’ medical data from Sutter Physicians Services were stolen [13]. These incidents highlight the importance of data integrity auditing protocol in cloud computing.

In light of the aforementioned context, this paper aims to enhance the data integrity verification process in a multi-cloud environment. Nevertheless, maintaining data integrity in a multi-cloud scenario presents several challenges. First, considering that stored data may have a large amount of size if the verifier has to download all the data in advance, it is both impractical and inefficient. Besides, the verifier may not have enough resources. The threat to users’ privacy is more significant and there is no assurance of unbiased data verification. Therefore, various kinds of research are conducted to develop auditing protocols in the cloud system. Particularly, research on verifying data integrity in cloud servers without requiring access to whole data is receiving much attention.

A pioneer in addressing this issue is Ateniese et al. [14]. The authors proposed an auditing protocol called Provable Data Possession (PDP). They introduce the concept of probabilistically checking the data integrity stored by users in the cloud server. Through this technique, users may be able to efficiently check the data integrity without saving the original data locally. The authors [15] proposed a scheme called Proof of Retrievability (PoR). They use spot-checking and error-correcting codes to guarantee data possession and retrievability on remote storage systems. Afterward, other data integrity verification protocols have been proposed in [3], but those protocols only focus on a single cloud environment.

The second challenge is unreliable verifiers. Some work explained in [4] includes a data integrity verification scheme in a multi-cloud environment. However, the scheme is inefficient because the verifier needs to check the data from each CSP separately [4,6]. Some authors proposed batch data integrity verification in a multi-cloud environment presented in [16]. Unfortunately, these approaches use a third-party auditor (TPA) and assume they are reliable, while, in reality, TPA may not be honest and it is difficult to find TPA trusted by multiple CSPs [3,6]. The development of blockchain technology is a promising solution for the above challenges. The decentralized and transparent nature of blockchain has become the main interest of the academic community to propose data integrity verification schemes. For example, Refs. [5,6,7] uses blockchain smart contracts to replace centralized TPA as a trusted verifier.

The third challenge is that most of the algorithms employed to audit data use a sampling method [3,4,5,6,7,14,17]. This means they randomly challenge some data blocks to provide probabilistic data possession proof. The probabilistic scheme relies on the spot-checking approach in which only a random fraction of the file is checked [4]. In a multi-cloud environment, users will store huge amounts of data. Thus, this paper wants to increase the sampling without increasing the cost by proposing a distributed data integrity verification scheme.

Motivated by the challenges mentioned above, to assure data integrity in a multi-cloud environment, this paper proposes a distributed data integrity verification in a multi-cloud environment by utilizing blockchain technology that also provides a batch verification process. This scheme will spread the verification task among several verifiers, thereby reducing computation and communication costs.

This paper’s main contributions are summarized as follows:

- This paper designs and proposes a blockchain-based distributed data integrity verification in the multi-cloud environment that increases the verification sampling rate without increasing the computation and communication costs by enabling data verification with multi-verifiers rather than single verifiers. By doing so, the burden is distributed to several verifiers.
- This scheme supports batch verification to increase the efficiency of proof validation and lower the cost for the cloud organizer (CO).
- This paper presents security and performance analyses of distributed data integrity verification protocols under the multi-verifier case. The security analysis consists of a proof of correctness of the equations and unforgeability by malicious CSPs, verifiers, and COs. The performance analysis consists of evaluating computation and communication costs, experiment results, gas usage estimation, and a comparative analysis of existing works.

The rest of the paper is organized as follows. This paper presents preliminaries in Section 3. Next, the property requirements for the data verification protocol are presented in Section 4. Further, the related work is provided in Section 2. This paper’s proposed scheme is presented in Section 5. This paper also presents a security analysis and performance evaluation in Section 6 and Section 7, respectively. Finally, the conclusion of the paper is presented in Section 8.

## 2. Related Works

The authors in [7] proposed a blockchain-based cloud data integrity verification using a lattice signature, cuckoo filter, and Merkle Hash Tree (MHT). The data are divided into blocks and a lattice signature is used to compute the tag for each data block. At the same time, the cuckoo filter is used for a lookup table in the verification process. However, in this paper, the signature of each data block is stored in the CSP. When users challenge CSP, CSP may only send previously stored signatures rather than recompute the proof from the file itself. So, the signatures will not represent the data integrity itself. Furthermore, this scheme did not support batch verification and concentrated on a single cloud environment. The authors of [5] proposed a blockchain-based cloud data integrity verification using the ZSS signature and T-Merkle tree. ZSS signature is used to compute the tag for each data block. They employ bilinear pairings in the verification process to support blockless verification and the T-Merkle tree is used as a data structure. However, this work did not support distributed verification by multi-verifiers and focused only on a single cloud environment.

The authors of [6] proposed a blockchain-based data integrity verification for multi-cloud storage. They use a homomorphic verifiable tag (HVT) to generate a tag for each data block. The verification process is performed through a homomorphism equation to support blockless verification. However, this work did not support distributed verification using multi-verifiers. The authors of [17] proposed a data integrity verification scheme for cloud storage using algebraic signatures. The verifier in this paper is a third-party auditor (TPA), which is assumed to be faithful and reliable. However, in reality, there is no guarantee for that claim. Therefore, this paper did not support a trusted verifier and only focused on a single cloud environment.

The authors of [18] proposed a blockchain-based public auditing scheme in multi-replica and multi-cloud environments. This work is based on a certificateless cryptosystem that aims to avoid the certificate management problem in PKI and key escrow problems in an identity-based cryptosystem. Unfortunately, this paper did not support batch verification and distributed verification. The authors of [19] proposed a blockchain-based public auditing scheme for cloud storage without trusted auditors. Their work replaced trusted third-party auditors (TPAs) with blockchain to resist malicious auditors. They also used certificateless public auditing to avoid key escrow problems. Unfortunately, this work did not support distributed verification and was only concentrated on a single cloud environment.

The authors of [20] used a BLS signature in their data integrity auditing protocol. They claimed that their scheme could efficiently reduce metadata computation costs for clients during the system setup phase in auditing protocol. However, this work did not support batch and distributed verification. The authors of [21] used an attribute-based signature in their data integrity auditing protocol. In this scheme, the users’ private keys are generated through arbitrary attributes chosen by users. By doing so, this signature enables data owners to specify the scope of auditors to avoid a single point of failure in traditional protocols, which have a single TPA. However, this work did not support batch verification and only focused on a single cloud environment.

The authors of [22] proposed a blockchain-based data integrity verification for large-scale IoT data. Instead of relying on trusted TPAs, this work used blockchain in the verification process. They also use HVT to generate tags for each data block. Unfortunately, this scheme did not support batch and distributed verification by multi-verifiers. The authors of [23] proposed a distributed machine learning-oriented data integrity verification scheme in a cloud computing environment. They adopted PDP as a sampling auditing algorithm in their scheme and generated a random number called a blinding factor and applied a discrete logarithm problem (DLP) to construct a proof and ensure privacy protection in the TPA verification process. However, this scheme did not support batch verification and distributed verification. This work also focused only on a single cloud environment. Furthermore, the verification uses a TPA that is assumed to be trusted. In reality, there is no guarantee for that claim.

From the above review of existing works, the challenge that is still ongoing is to accomplish batch verification and provide a distributed data verification process using multi-verifiers. Therefore, this paper proposes a blockchain-based distributed data integrity verification in a multi-cloud environment that provides a batch verification process. This paper aims to increase the sampling rate without increasing the costs by enabling the verification process to be performed by multi-verifiers rather than only a single verifier. Furthermore, batch verification will increase efficiency and decrease costs during the verification process for the cloud organizer in a multi-cloud environment.

## 3. Preliminaries

### 3.1. Bilinear Pairings

Let $G_{1}$ be a cyclic additive group and $G_{2}$ be multiplicative cyclic groups with a prime order *p*; *P* is the generator of the group $G_{1}$. The mapping $e:G_{1}\times G_{2}\to G_{2}$ is a bilinear map with the following properties:

1. Bilinearity: $e\left(aP,bQ\right)=e{\left(P,Q\right)}^{ab}$, and e(P+R,Q)=e(P,Q)·e(R,Q), for all P,Q,$R\in G_{1}$, $a,b\in Z_{p}.$
2. Computability: There is an efficient algorithm to compute e(P,Q), for all $P,Q\in G_{1}$.
3. Non-degeneracy: There exists $P\in G_{1}$ such that e(P,P)≠1.

This paper considers the following problems in the additive group $G_{1}$.

- Discrete Logarithm Problem (DLP): Given two group elements P and Q, find an integer $n\in Z_{p}^{*}$, such that Q=nP whenever such an integer exists.
- Computational Diffie–Hellman Problem (CDHP): For $a,b\in Z_{p}^{*}$, given P,aP,bP, compute abP.

There are two variants of CDHP:

- Inverse Computational Diffie–Hellman Problem (Inv-CDHP): For $a\in Z_{p}^{*}$, given P,aP, compute $a^{-1}P$.
- Square Computational Diffie–Hellman Problem (Squ-CDHP): For $a\in Z_{p}^{*}$, given P,aP, compute $a^{2}P$.

### 3.2. ZSS Signature

ZSS is a short signature based on bilinear pairing first proposed by Zhang, Safavi-Naini, and Susilo [24]. The main idea is to construct a signature that is a difficult CDH problem in a group *G*. This signature required less pairing operation than other short signatures, such as the Boneh–Lynn–Shacham (BLS) signature, causing it to be more efficient [24]. There are four steps in the ZSS signature:

1. ParamGen. Let $G_{1}$ be a cyclic additive group and $G_{2}$ be multiplicative cyclic groups with a prime order *q*, *P* is the generator of the group $G_{1}$. The mapping $e:G_{1}\times G_{2}\to G_{2}$ is a bilinear map. *H* is a general hash function. So, the system parameters are $\left\{G_{1},G_{2},e,q,P,H\right\}$.
2. KeyGen. Randomly selects $x\in_{R}Z_{q}^{*}$ and computes $P_{pub}=xP$. The public key is $P_{pub}$.
3. Sign. Given a secret key *x* and a message *m*, computes signature $S={\left(H(m)+x\right)}^{-1}P$.
4. Ver. Given a public key $P_{pub}$, a message *m*, and a signature *S*, verify if $e(H\left(m\right)P+P_{pub},S)=e\left(P,P\right)$. The verification works because of the following Equation (1).
  (1) $$\begin{array}{cc} e(H\left(m\right)P+P_{pub},S) & =e(\left(H\left(m\right)+x\right)P,{\left(H\left(m\right)+x\right)}^{-1}P)\\ \; & =e{\left(P,P\right)}^{\left(H\left(m\right)+x\right)\cdot {\left(H\left(m\right)+x\right)}^{-1}}\\ \; & =e(P,P) \end{array}$$

## 4. Properties Requirements

This section presents the design goal of our proposed scheme. In [3], the authors have defined some desirable properties for data integrity auditing protocols. This paper adopts some of their properties as follows.

- Public verifiability: the size of the stored data may vary and there is a possibility that users’ data are large. Furthermore, users may have limitations on their resources that can cause an expensive verification process cost. Therefore, the verification mechanism should not only allow users that can verify the data but also allow other parties.
- Blockless verification: stored data size varies and the verifier may not have enough resources to process a large amount of data. The verification scheme must ensure the verifier does not need to download all the data for the verification process. Furthermore, it will decrease the communication overhead at the server and increase the efficiency of the verification scheme.
- Privacy-preserving: prevent data leaks to the verifier during the verification process.
- Batch verification: decreases communication costs for the verifier and the cloud server.

Nevertheless, besides some requirements from [3] above, we consider it crucial to have a reliable verifier and cloud organizer in multi-cloud environments. Since private verification methods fail to provide mutual trust in verification results [3,6], public verification methods are introduced. In public verification, users delegate TPA to validate the stored data in CSP. However, protocols that use a TPA as a verifier assume that TPA is faithful and reliable [6]. In reality, TPA may become untrustworthy and collude with other parties to manipulate users. Therefore, this paper also adds three other requirements as follows.

- Reliable verifiers: verifiers play a significant role in data integrity verification schemes. The results will determine the trustability of a CSP as a data host for many users. It is important to ensure that there is no way the verifier could collude with the CSP to deceive users or with users to deceive the CSP. For instance, it can be achieved by performing a decentralized verification process to provide transparency.
- Reliable cloud organizer (CO): in a multi-cloud environment, the cloud organizer manages the interaction between the user and CSPs [6]. It is important to ensure the organizer sends the right data to the intended CSP. Likewise, send the correct proof to the verifier during the verification process.
- Distributed verification: under the case of the multi-cloud environment, stored users’ data may be huge. It is necessary to increase the verification sampling rate without escalating the costs required on the verifier’s side. One way to perform that is by enabling distributed verification to multi-verifiers. Doing so will distribute the burden of verification tasks and not solely rely on one verifier.

## 5. Proposed Scheme

This section presents the distributed data integrity verification scheme in the multi-cloud environment that supports the aforementioned requirements in Section 4. This proposed protocol has four types of actors: user, CO, CSP, and verifier. The user is the one that stores the data in multiple CSPs and wants to verify the integrity of the stored data. Since it is a multi-cloud environment, a CO controls the interaction between users and CSPs [6]. The CO will send the user’s data to each CSP. They will also assign the verifiers when the user requests data integrity verification. Each verifier will send a challenge request to the corresponding CSP related to the data that need to be verified. Later, the CSP will reply by sending proof of so-called δ to the corresponding verifier. The verifier will verify each proof with a bilinear pairing equation by generating a proof of the so-called ω as the pair of proof δ. In the final step of the verification process, the CO will aggregate proofs from the verifiers and perform batch verification.

**Figure 1:**
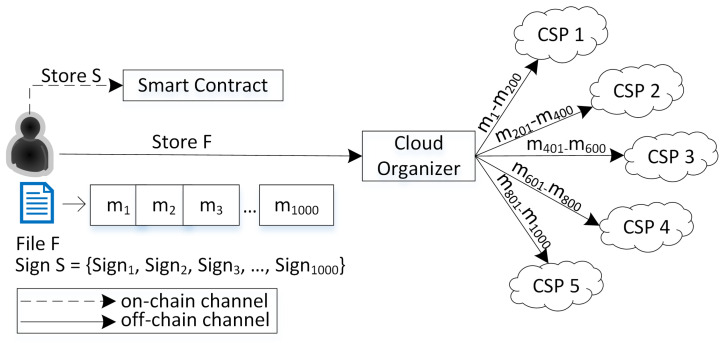
Scenario example of the proposed scheme.

This paper presents a numerical example of the proposed scheme through Figure 1.

The user splits the file *F* into 1000 blocks in the example. So, the user will generate 1000 ZSS signatures (*S*). This paper chose the ZSS signature because it required less pairing operation than other short signatures, such as the BLS signature [24]. Furthermore, ZSS does not need a special hash function, i.e., MapToPoint, when used in BLS. So, we can use a general hash function such as SHA family or MD5 [24]. After that, the user sends file *F* to CO and signs *S* to the smart contract. Then, CO distributes 1000 blocks into several CSPs. Assuming that the CO spreads to five CSPs, each CSP will store 200 data blocks. The CO also stores the list of block ranges stored in each CSP in its local database. Then, in the verification phase, if the CO assigns four verifiers, each verifier can verify 250 data blocks. When assigning the verifier, the CO will also send the information of the CSPs that stored the related data. In this case, because each CSP only stores 200 data blocks earlier, each verifier needs to send challenges to two CSPs to obtain the rest of the data blocks. For example, verifier-1 will send challenges to CSP 1 for blocks 1–200 and CSP 2 for blocks 201–250. Verifier-2 will send challenges to CSP 2 for blocks 251–400 and CSP 3 for blocks 401–500. This pattern continues until the fourth verifier. Therefore, each verifier will have two proofs δ. Subsequently, each verifier will generate proof ω as a pair for each proof δ. So, in this example, there will be eight pairs of proofs δ and ω. In the end, CO will aggregate these eight pairs to perform a batch verification and send the result to the user.

This paper provides a list of notations used in the proposed scheme in Nomenclature and explain the details of the proposed protocol as follows.

**Figure 2:**
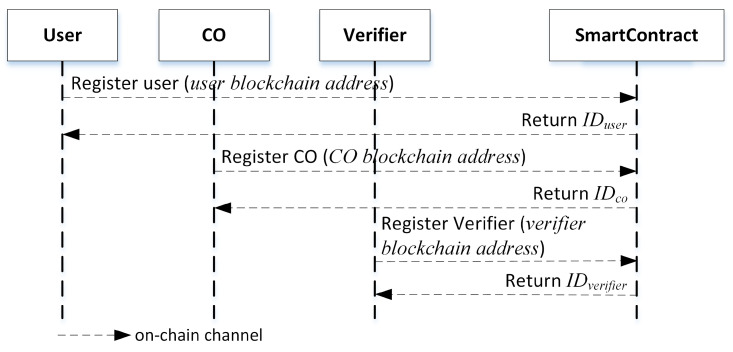
Registration phase.

**Figure 3:**
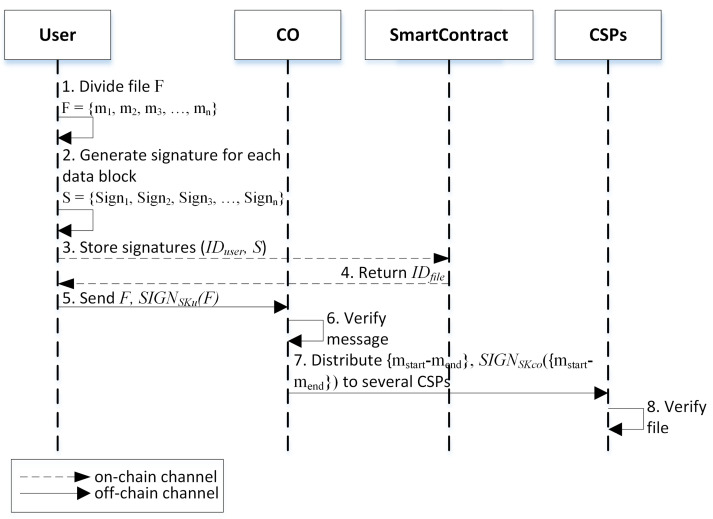
Data storing phase.

**Figure 4:**
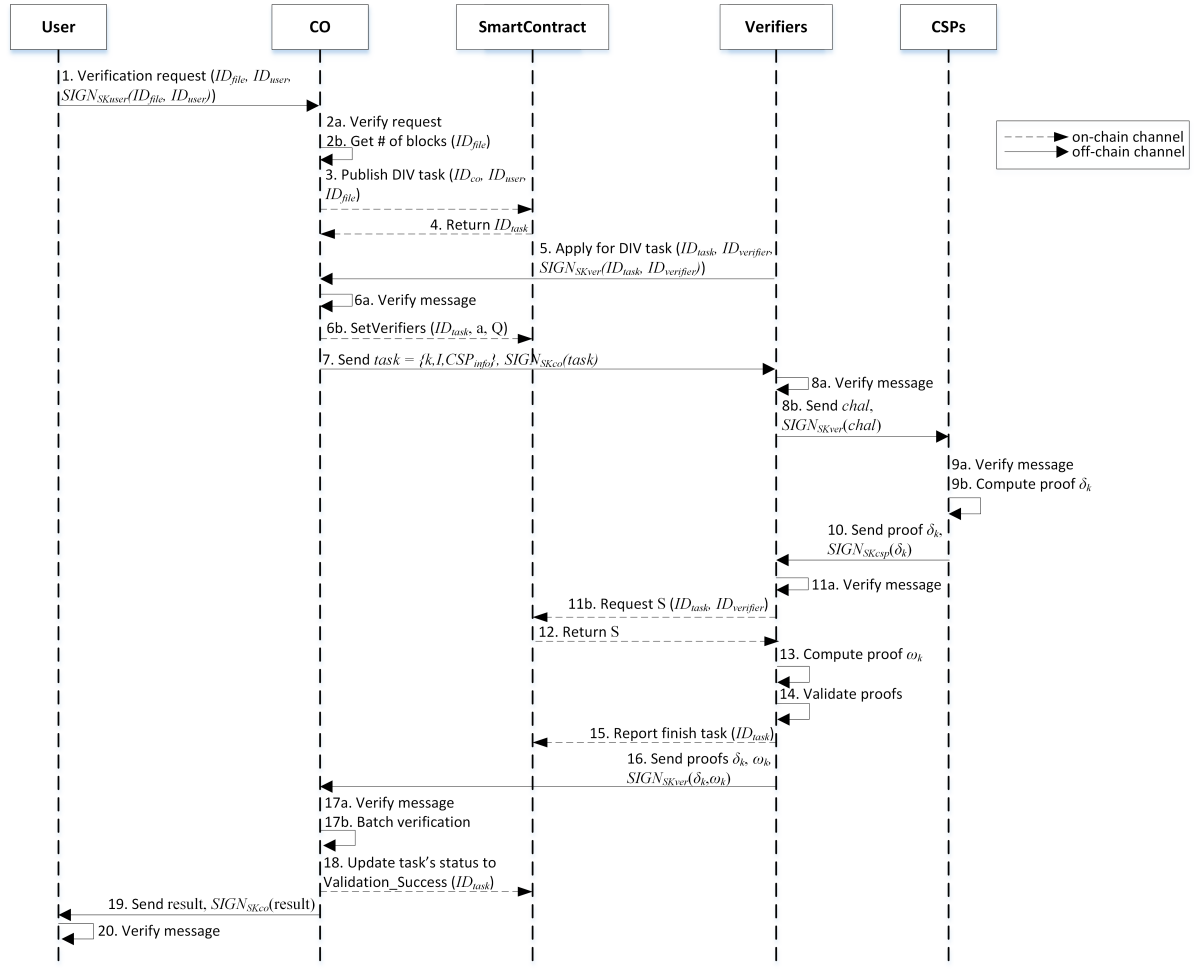
Data verification phase.
  9. (a.) Upon receiving chal from the verifier, the CSP verifies the received message’s signature first using the verifier’s public key. If valid, continue; otherwise, reject it. (b.) Afterward, the CSP will compute the proof $\delta_{k}$ as shown in Equation (3). In the above scenario, for verifier-1, CSP 1 generates $\delta_{1}$ blocks 1–200 and CSP 2 generates $\delta_{2}$ blocks 201–250, respectively.
    (3) $$\delta_{k}=\sum_{i\in I}r_{i}\left(H\left(m_{i}\right)P+P_{pub}\right)$$
  10. Later, the CSP sends proof $\delta_{k}$ to the corresponding verifier along with the signature of the message that is signed with the CSP’s private key ($SK_{csp}$) to the verifier. So, based on the given scenario, verifier-1 will receive $\delta_{1}$ from CSP 1 and $\delta_{2}$ from CSP 2.
  11. (a.) The verifier then verifies the received message’s signature using the CSP’s public key. If valid, continue; otherwise, reject it. (b.) Next, the verifier requests the ZSS signature *S* value to the smart contract according to the ranges of data blocks to be verified. So, based on the scenario above, verifier-1 will request *S* for blocks 1–250.
  12. In response to the verifier, the smart contract sends the corresponding *S* value.
  13. Subsequently, the verifier computes proof $\omega_{k}$. The $\omega_{k}$ is the pair of the proof $\delta_{k}$. So, in Equation (4), the value of a set *I* is the same as *I* in Equation (3) for the same *k*. From the given scenario above, verifier-1 generates $\omega_{1}$ blocks 1–200 and $\omega_{2}$ blocks 201–250.
    (4) $$\omega_{k}=\sum_{i\in I}r_{i}Sign_{i}$$
  14. After that, the verifier checks the validity of each pair of proofs $\delta_{k}$ and $\omega_{k}$ as shown in Equation (5). So, from the example above, verifier-1 will perform the bilinear pairing for proofs $\delta_{1}$,$\omega_{1}$ and $\delta_{2}$,$\omega_{2}$, respectively. If the equation holds, the data blocks in the corresponding CSP are safe; otherwise, the verifier will know which CSP failed to prove the integrity of the user’s data.
    (5) $$e\left(\delta_{k},\omega_{k}\right)=e\left(P,P\right)$$
  15. Consequently, the verifier reports the results to the smart contract.
  16. Then, each verifier sends proofs $\delta_{k}$, $\omega_{k}$ and the signature of the message signed with the corresponding verifier’s private key to the CO.
  17. (a.) In this step, the CO will verify the received message’s signature using the verifier’s public key. If valid, continue; otherwise, reject it. (b.) Next, in this step, the CO receives several proofs δ and ω from multiple verifiers and will start the batch verification. In the above example, there are four verifiers. The batch verification in this work supports aggregating proofs of file *F* from multi-verifiers. In the process, the CO will check if Equation (6) holds, where *K* is a set of proofs = {1,2,3,…,t} and *t* is the total number of proofs that the CO received from verifiers. To calculate *t*, first, let v= the number of blocks stored in the CSP and a= number of verifiers. If vmodc equals 0, then t=a. Otherwise, check if v>c, then t=⌈v/c⌉×a; otherwise, t=⌈c/v⌉×a. In the given scenario, the CO receives eight pairs of proofs δ and ω.
    (6) $$e\left(\sum_{k\in K}\delta_{k},\sum_{k\in K}\omega_{k}\right)=e\left(P,P\right)$$
  18. If it holds, update the verification task’s status in the smart contract to success; otherwise, it failed.
  19. The CO reports the DIV result to the corresponding user and the message’s signature that is signed with CO’s private key ($SK_{co}$).
  20. After receiving the result from the CO, the user will verify the signature using the CO’s public key. If valid, the user is assured that the message is indeed from the CO; otherwise, it rejects it.

- Setup phase.
  - ParamGen. Let $G_{1}$ be a Gap Diffie–Hellman (GDH) group and $G_{2}$ be multiplicative cyclic groups with prime order *q*; *P* is the generator of a group $G_{1}$. The mapping $e:G_{1}\times G_{1}\to G_{2}$ is a bilinear map with some properties. *H* is a general hash function such as the SHA family or MD5. The system parameters are $\left\{G_{1},G_{2},e,q,P,H\right\}$.
  - KeyGen. Randomly selects $x\in Z_{q}$ as a secret key and computes $P_{pub}=xP$ as a public key.
  - User generates ZSS signature for each data block $m_{i}$ as shown in Equation (2).
    (2) $$Sign_{i}={\left(H\left(m_{i}\right)+x\right)}^{-1}P$$

- Registration Phase.
  In the beginning, each user, CO, and verifier need to register to the smart contract once by sending their blockchain address as shown in Figure 2. After that, they will receive an ID so-called $ID_{user},ID_{co},$ and $ID_{verifier}$, respectively, that will be used in the storing and verification phases. These IDs are assigned by smart contract internal generation rules. Each ID starts with a different first digit to determine the category. The first digit for $ID_{user}$ is 1, $ID_{co}$ is 2, and $ID_{verifier}$ is 3, while the rest digit shows the serial number of the ID.

- Storing Phase.
  The details of the data storing phase are shown in Figure 3 as follows.
  1. In this phase, the user divides the file *F* into *n* blocks of *m*. For the general case, let each block size be 4 KB. So, the file $F=\{m_{1},m_{2},m_{3},\ldots ,m_{n}\}$.
  2. Then, he will generate a signature Sign for each data block. So, the set of signatures is $S=\{Sign_{1},Sign_{2},Sign_{3},\ldots ,Sign_{n}\}$.
  3. Subsequently, the user stores the signatures in the smart contract by sending the $ID_{user}$ and *S* values.
  4. Then, the smart contract will assign an $ID_{file}$ value, a unique identity number for each file *F*, to the user. $ID_{file}$ is assigned by the smart contract internal generation rule that begins with 4 as the first digit. The rest of the digits show the serial number of the $ID_{file}$.
  5. Next, the user sends file *F* and also the signature of *F* that is signed with the private key of the user ($SK_{u}$) to the CO.
  6. Upon receiving *F* from the user, CO verifies the signature first using the user’s public key. If valid, CO continues the process; otherwise, it rejects it. The signature verification process is important to prove that the sender is the real user and to prevent the adversary from impersonating the real sender.
  7. The next process involves CO distributing it to several CSPs with ranges $m_{start}$ showing the beginning of the data blocks, while $m_{end}$ shows the last data blocks that will send to the CSP. The CO also sends along the digital signature of the message that is signed with the CO’s private key ($SK_{co}$). Subsequently, the CO stores the list of data blocks and the corresponding CSPs in the local database.
  8. The CSP will verify the file from the CO using the CO’s public key. If valid, store the file; otherwise, reject it.

- Verification Phase.
  The details of the data verification phase are shown in Figure 4 as follows.
  1. The user sends a data integrity verification (DIV) request by transmitting $ID_{file}$, $ID_{user}$, and the signature of the corresponding message that is signed with the user’s private key ($SK_{u}$) as parameters to the CO.
  2. (a.) Then, CO verifies the signature of the message received from the user using the user’s public key. (b.) If valid, it will obtain the number of blocks of the corresponding $ID_{file}$; otherwise, the CO rejects it. The signature verification process is important to prove that the sender is a real user and also prevents the adversary from impersonating the real sender.
  3. After that, the CO publishes a DIV task in the smart contract by sending $ID_{co}$, $ID_{user},$ and $ID_{file}$ as parameters.
  4. The smart contract processes the request and assigns a $ID_{task}$ to be returned to the CO, which is a unique number for each task.
  5. Since it is a broadcast message, all the nodes that join the blockchain network will receive this notification. Several verifiers will then apply to perform the verification task for the CO by sending the corresponding $ID_{task},ID_{verifier}$, and the signature of the message that is signed by the verifier’s private key ($SK_{ver}$).
  6. (a.) Subsequently, CO verifies the message from the verifier with the verifier’s public key. If valid, continue; otherwise, reject it. (b.) Afterward, CO sets the verifiers that will perform the DIV task by sending $ID_{task},a,$ and *Q* as parameters to the smart contract, where *a* is the number of verifiers and *Q* is the set of $ID_{verifier}$ assigned to perform a verification task.
  7. The CO then sends $task=\{k,I,CSP_{info}\}$ and the signature of the task that is signed with CO’s private key ($SK_{co}$) to each selected verifier, where *k* is an index of the proof that will be generated by the corresponding CSP, *I* is the set of challenged data blocks so I={1,2,3,…,c}, and *c* is the total number of challenged data blocks for each CSP. $CSP_{info}$ contains the corresponding CSP information. So, in the given scenario, the CO will send two task to verifier-1, first with k=1, *I* with c=200 blocks, and $CSP_{info}$ of CSP 1. Second, k=2, *I* with c=50 blocks and $CSP_{info}$ of CSP 2.
  8. (a.) After receiving task from the CO, the verifier will verify the signature of the received message using the CO’s public key. If valid, continue; otherwise, reject it. (b.) Then, each verifier will send challenge $chal={\left\{k,i,r_{i}\right\}}_{i\in I}$ and the signature of the message that is signed with verifier’s private key ($SK_{ver}$) to the corresponding CSPs that have data blocks needed to be verified, where $r_{i}$ is a random number in $Z_{q}$ and *i* is an index of randomly selected data blocks to be verified in the set of *I*.

## 6. Security Analysis

### 6.1. Correctness

Given the proofs, $\delta_{k},\omega_{k}$, the verifier can correctly check the integrity of the challenged user’s data blocks stored in the CSP. Based on the properties of bilinear pairings explained in Section 3.1, the correctness of Equation (5) can be proved as follows.

(7) $$\begin{array}{cc} e(\delta_{k},\omega_{k}) & =e(\sum_{i\in I}r_{i}\left(H\left(m_{i}\right)P+P_{pub}\right),\sum_{i\in I}r_{i}Sign_{i})\\ & =e(\sum_{i\in I}r_{i}\left(H\left(m_{i}\right)+x\right)P,\sum_{i\in I}r_{i}{\left(H\left(m_{i}\right)+x\right)}^{-1}P)\\ & =e{\left(P,P\right)}^{\sum_{i\in I}r_{i}\left(H\left(m_{i}\right)+x\right)\cdot \sum_{i\in I}r_{i}{\left(H\left(m_{i}\right)+x\right)}^{-1}}\\ & =e(P,P) \end{array}$$

Given the proofs, $\delta_{k},\omega_{k}$, from the verifiers, CO can correctly check the validity of the challenged user’s data blocks stored in the CSP in batch verification. Based on the properties of the bilinear pairings explained in Section 3.1, the correctness of Equation (6) can be proved as follows.

(8) $$\begin{array}{cc} e(\sum_{k\in K}\delta_{k},\sum_{k\in K}\omega_{k}) & =e(\sum_{k\in K}\sum_{i\in I}r_{ki}\left(H\left(m_{ki}\right)P+P_{pub}\right),\sum_{k\in K}\sum_{i\in I}r_{ki}Sign_{ki})\\ & =e(\sum_{k\in K}\sum_{i\in I}r_{ki}\left(H\left(m_{ki}\right)+x\right)P,\sum_{k\in K}\sum_{i\in I}r_{ki}{\left(H\left(m_{ki}\right)+x\right)}^{-1}P)\\ & =e{\left(P,P\right)}^{\sum_{k\in K}\sum_{i\in I}r_{ki}\left(H\left(m_{ki}\right)+x\right)\cdot \sum_{k\in K}\sum_{i\in I}r_{ki}{\left(H\left(m_{ki}\right)+x\right)}^{-1}}\\ \; & =e(P,P) \end{array}$$

### 6.2. Unforgeability

This part presents the unforgeability of the verification results in the proposed scheme from the attackers. There are three cases as follows.

1. A malicious CSP may lose the user’s stored data and try to hide it from the users or delete the rarely accessed user’s data to reduce the storage burden. So, when a malicious CSP receives a challenge from the verifier in the verification process, it may try to forge proof δ to deceive the verifier.
2. A malicious verifier may collude with CSP to deceive the user/CO or with the user/CO to deceive the CSP. So, this untrusted verifier may try to forge the proof ω to manipulate the verification results.
3. A malicious CO may collude with the verifier to deceive the user or collude with the user to deceive the verifier. Therefore, the malicious CO may try to forge the validation results of aggregation proofs from verifiers.

Based on the above three cases, we prove the unforgeability of each case as follows.

- A malicious CSP cannot forge proof δ because every time the verifiers send a challenge, a random value *r* will be assigned in the chal variable. The CSP must generate proof δ based on *r* from the verifiers as shown in Equation (3) and the value *r* is different for each data shard. Even if we further assume that the malicious CSP forges δ, namely $\delta \neq \delta^{\prime }$, the verification process performed by the verifiers in Equation (5) shows that the verifiers must validate $\delta^{\prime }$ with another variable, proof ω generated by verifiers, which also consist of a *r* value. Therefore, the $\delta^{\prime }$ cannot ensure Equation (5) will hold. Another case is when a malicious CSP tries to deceive the verifier by replacing the challenged data block $m_{j}$ with another data block $m_{b}$ when the former data block is broken. Accordingly, the proof $\delta^{\prime }$ becomes
  (9) $$\delta^{\prime }=\sum_{i\in I,i\neq j}r_{i}H\left(m_{i}\right)P+r_{j}H\left(m_{b}\right)P$$
  So, Equation (5) can be represented as
  (10) $$e\left(\delta^{\prime },\omega \right)=e\left(\delta ,\omega \right)=e\left(P,P\right)$$
  Hence, we have $H\left(m_{b}\right)=H\left(m_{j}\right)$. However, $H(m_{b})$ cannot be equal to $H(m_{j})$ due to the anti-collision property of the hash function. Therefore, it is infeasible to ensure Equation (10) will hold and the proof from CSP cannot pass the verification process.
- A malicious verifier cannot forge verification results because the verifier must generate proof ω that requires Sign, which is a signature generated by the user as shown in Equation (4). The Sign values are stored in the smart contract, which is very difficult to be tampered with. Even we further assume that the malicious verifier forges proof ω to generate variable Sign, the malicious verifier needs the user’s secret key *x* and original message $m_{i}$. The proposed scheme provides privacy-preserving and blockless verification properties, meaning the verifier is capable of verifying the data without receiving the original message $m_{i}$ from the user or CSP. So, without the possession of those two variables, *x* and $m_{i}$, it is impossible to generate a forged $m_{i}^{\prime }$ and enable equation ${\left(H\left(m_{i}^{\prime }\right)+x\right)}^{-1}P={\left(H\left(m_{i}\right)+x\right)}^{-1}P$ to hold. Both the malicious CSP and verifier cannot calculate the user’s private key from the public key under the Inv-CDHP assumption. Furthermore, in the verification process, the verifier must validate ω with another variable, proof δ generated by CSP as shown in Equation (5). Therefore, it is infeasible to cause equation $e\left(\delta ,\omega^{\prime }\right)=e\left(P,P\right)$ to hold where $\omega^{\prime }\neq \omega$.
- A malicious CO cannot forge validation results because he needs to aggregate several proofs from verifiers and CSPs as shown in Equation (6). As explained above, proof δ is generated by CSPs and proof ω is generated by the verifiers. Each proof required a random value *r* that was generated randomly by each verifier as shown in Equations (3) and (4). In addition, these values did not send to the CO in the protocol. Therefore, the CO will not cause Equation (6) to hold.

## 7. Performance Evaluation

This section presents the proposed protocol’s implementation results and evaluates the performance by calculating the estimation of gas consumption, computation, and communication costs.

The prototype was implemented using Docker installed on Windows 10 with Intel Core i5-7200U @2.50 GHz CPU and 4 GB memory. There are four main actors, user, CO, CSP, and verifier, with the specifications shown in Table 1. The blockchain network was based on Ganache. Ganache is a rapid Ethereum and Corda distributed application development [25]. The smart contract is written in the Solidity programming language. The ZSS signature generation code was implemented based on the Pairing-Based Cryptography (PBC) Go Wrapper package, while for the digital signature, this paper uses the crypto-ecdsa package with the Go language version 1.19.3 [26,27]. The parameters of PBC that this paper used are type A param, with group order 160 bits and the base field order 512 bits.

### 7.1. Computation Cost

The computation cost for the proposed protocol is shown in Table 2. The result for the user is (n×(Inv+Add+Mul+Hash))+2SIGN+VER with the front bracket showing the cost for generating ZSS signatures for *n* data blocks. The cost of the CSP is (c×(2Mul+Add+Hash))+SIGN+2VER with the bracket showing the cost for generating proof δ, while the cost of the verifier is ((c×Mul)+(c×P))+2SIGN+2VER with the bracket showing the cost for generating proof ω and bilinear pairing of proofs δ,ω in the verification process. The last is the cost of CO, ((t×Add)+P)+3SIGN+3VER with the bracket showing the cost for the batch verification process.

Furthermore, a variable *c* shows the number of data blocks that need to be verified by each verifier. This variable depends on the total number of data blocks (*n*) and the total number of verifiers (*a*). So, based on the given scenario in Section 5, if *n* is 1000 blocks and *a* is 1, *c* is 1000. It means one verifier needs to verify all 1000 data blocks. As a result, the computation cost will be higher since its calculation will grow linearly as the number of *c* increases. In the case of 1 user with multi-verifiers, if *a* is 4, so each verifier must verify 250 data blocks. As a result, the computation cost will be lower because the burden is distributed to four verifiers. The proposed work also supports batch verification for the CO. At the end of the verification process, the CO will receive several proofs δ and ω from multi-verifiers. Instead of verifying one by one, the CO will aggregate those proofs and only perform bilinear pairing once.

This paper also presents the average computation time results for each operation used in the proposed scheme in Table 3. The results show that multiplication and bilinear pairing operations consumed the highest average time, 1.464 ms and 1.125 ms, respectively. In comparison, addition and hash operations have the lowest computation time, 0.0001 and 0.0007 ms, respectively.

### 7.2. Communication Cost

This paper presents the communication cost for each actor as shown in Table 4. Those costs are calculated from the size of the data transferred (bytes) between the actors in the proposed protocol. The communication cost for the user is calculated from the storing data in the CSP that costs (n×4000)+128 and sends a verification request to the CO, which costs 134 bytes. The size of the digital signature in the proposed scheme is 128 bytes generated by the crypto-ecdsa Go package. So, based on the given scenario in Section 5 with n=1000, the total communication cost of the user is 4,000,262 bytes or around 4 MB. The user seems to have a high communication cost in the storing phase depending on the data blocks the user will store, but it will only be performed once. For the CSP, the communication cost is for sending the generated proof δ along with the digital signature to the verifier, which costs 218 bytes.

The verifier’s communication costs are calculated from sending challenges to the CSP and sending proofs δ,ω to the CO, which are (c×47)+128 and 308 bytes, respectively. So, from the given scenario, if using one verifier, a=1,c=1000, the total cost becomes 47,436 bytes, while, in the case of multiple verifiers, if a=4,c=250, the total cost reduces to 12,186 bytes. In the library that this paper used, the PBC Go wrapper support function called compressedBytes() was used to convert a value to the compact bytes size. Therefore, the size of the generated proof δ or ω is 90 bytes. Lastly, the communication costs for the CO are accumulated from sending task to each verifier and sending verification result to the user, which are (a×(c+200))+128 and 133 bytes, respectively. So, from the presented scenario, the total communication costs for the CO if a=1,c=1000 is 1461 bytes, and 2061 bytes if a=4,c=250. The cost is slightly higher for the CO in the case of multi-verifiers. However, the trade-off is that it can reduce the costs for each verifier.

### 7.3. Experiment Results

**Figure 5:**
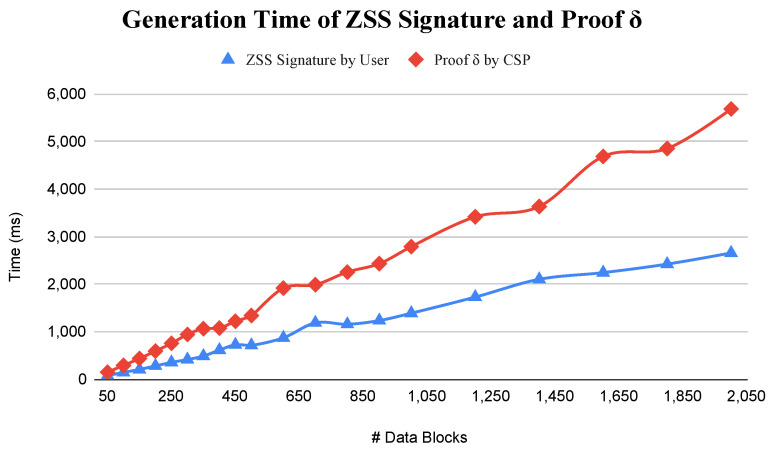
Generation time of Signature and Proof Delta.

In the experiments, each data block size is 4 KB. The minimum number of data blocks is defined as 50 and the maximum is 2000 data blocks. So, the minimum file size is 200 KB and the maximum is 8000 KB. In Figure 5, this paper presents the result of the generation time comparison of the ZSS signature by the user and proof δ by the CSP. It shows that the user and CSP generation time increases linearly with the CSPs with higher time consumption. The CSP reaches time 5.6 s for generating proof δ of 2000 data blocks and the user 2.6 s for generating the ZSS signature of the same amount of data blocks. The CSP needs a longer time because, as shown in Equation (3), it needs two multiplication operations. Different from the user that only needs one multiplication operation in Equation (2). Based on the average computation time for each operation in Table 3, the multiplication operation consumed the highest computation time compared tp the other operations, while the hash, inverse, and addition operations are negligible. So, the proposed scheme offers the users lower computation costs because they generally have limited resources compared to the CSP.

Next, this paper compares the file size of the total data blocks and generated ZSS signature. The result shows that even though the size of the data blocks increases linearly, the size of the ZSS signature is almost constant. For example, the 50 data block size is 200 KB and the ZSS signature size is 4.5 KB with the same number of blocks, while the 2000 data block size is 8000 KB and the ZSS signature size is only 182 KB. The generated ZSS signature size is smaller than the original data block because the ZSS signature processes the hash of the data rather than the original data. Therefore, the high reduction in size means it can reduce the overhead storage and communication costs on the user’s side, so they do not need to store other big-size files.

**Figure 6:**
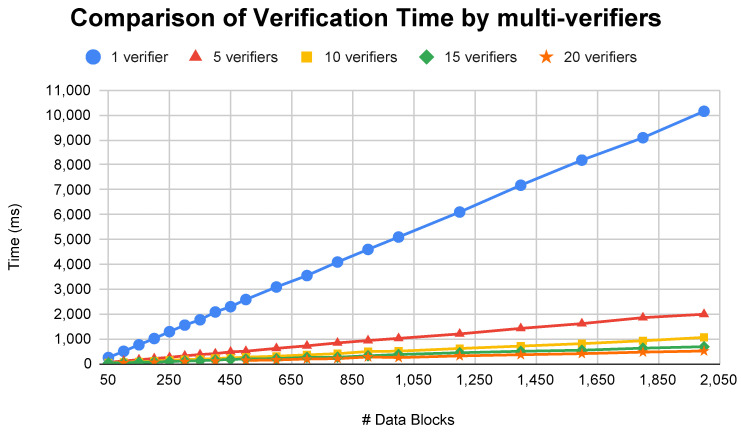
Comparison of verification time using multi-verifiers.

This paper also presented a simulation of multi-verifiers in the data verification process shown in Figure 6. This paper tested the performance by comparing the required verification time between one verifier and multi-verifiers. It is worth noting that the verification time presented omits the transmission time needed between verifiers, CSPs, and CO because the time will vary and be influenced by many factors. The results show that, in the case of one verifier, the time increases linearly alongside the increasing number of data blocks. It needs 10 s to verify 2000 data blocks. However, the case of multi-verifiers (5, 10, 15, and 20 verifiers) significantly reduces the time consumption with results of 1.9 s, 1 s, 0.6 s, and 0.5 s, respectively, for the same amount of data blocks.

Based on the results of the experiments, we can argue that the proposed protocol has the advantage of requiring less storage space, computation, and communication costs from the user, which also eliminates the need for them to keep additional large files. Furthermore, this paper proves that the proposed scheme can complete verification tasks faster by spreading the workload among multiple verifiers. Hence, reduce the costs required for the verifiers.

### 7.4. Gas Usage Estimation Cost

Table 5 shows the proposed protocol’s estimation of gas usage and fee in USD. Every user, verifier, and CO needs to register themselves for the first time to the smart contract; it costs 50,733 gas for each function number 1–3. Then, every time the users add their data to be verified, it costs 107,399 gas. In order to perform a verification task, the CO needs to run the AddTask function, which costs 171,257 gas. When the CO wants to assign verifiers for a verification task, he calls AssignVerifier at once to assign *a* number of verifiers, which costs 179,948 gas. The last function, SetTaskState, is able to be run by the CO or verifier to update the verification task status, which costs 32,141 gas.

According to the aforementioned analysis, we can conclude that, regardless of the number of verifiers, whether one verifier or multi-verifiers, the gas usage that the CO needed to perform the verification task is the same. However, the merit point of the proposed distributed data integrity verification protocol is highlighted in the computation and communication costs. In the case of one user − one verifier, the computation and communication costs will be higher because one verifier is required to verify all the data blocks. In contrast, the proposed protocol offers low computation and communication costs because, by utilizing more verifiers, the burden will be distributed evenly for each verifier. Furthermore, using batch verification, this paper also reduces costs for the CO.

### 7.5. Comparative Analysis

This section presents a comparative analysis between the proposed protocol and other related works, as shown in Table 6 below. The comparison points are based on the property requirements explained in Section 4. However, this paper omits the first three requirements, public verifiability, blockless verification, and privacy-preserving, because the proposed scheme and other related works already fulfilled those points. So, the main comparison points are batch verification, multi-cloud, reliable CO, reliable verifiers, blockchain-based, and distributed verification.

- Batch verification. The proposed protocols support batch verification as shown in Equation (6) where the CO will validate proofs from the verifiers. Compared to other work, two out of ten protocols also support batch verification, which is [6,19]. However, eight other protocols only verify the proof from CSPs one by one for each data block.
- Multi-cloud environment and reliable CO. The proposed protocols [6,18] provide data verification in a multi-cloud environment. In addition, in a multi-cloud environment, a CO is assigned to distribute the files from the user to several CSPs. The proposed protocols and the other two supported reliable CO by utilizing blockchain technology. Unfortunately, eight other protocols did not. They mostly focused on verification in a single cloud environment and no CO was needed.
- Reliable verifiers and blockchain-based. The proposed scheme and six out of ten other works support reliable verifiers by employing blockchain technology. This paper can design a decentralized verification process through blockchain to provide transparency between users and CSPs. It removes the intermediary and enables peer-to-peer interactions between nodes, therefore enhancing trust. The other four works [17,20,21,23] did not provide these two points because they rely on the credibility of the third-party auditor (TPA), which is not ideal in real case circumstances.
- Distributed verification. The proposed protocol is the only one that can accomplish it. The other ten protocols were unable to. This paper fulfills it by enabling multi-verifiers to participate in a verification task, whereas the other protocols were interested in data verification by a single verifier. This paper also has performed the verification simulation using 5, 10, 15, and 20 verifiers and demonstrated that the proposed scheme could complete the verification tasks faster with less computation and communication costs than the single verifier.

Based on these comparative studies, this paper concludes that most related works have supported reliable verifiers by employing blockchain technology. However, only a few works provide batch verification and support data verification in a multi-cloud environment. Lastly, the proposed scheme fulfills all the comparison points with the distributed verification, which becomes the most prominent benefit of the proposed protocol and is not owned by any other related work. As presented early in this section, the advantage of the proposed distributed verification is that this paper can reduce the computation and communication costs because the workload is evenly distributed among multiple verifiers.

### 7.6. Case Study

In the previous sections, this paper already outlined the proposed scheme and performance evaluations. This part provides a case study of the applications of the proposed distributed data integrity protocol.

Distributed machine learning: In distributed machine learning, several nodes are used to perform the training in parallel. Thus, it can increase scalability and optimize time. The training data are stored in different storage locations with the nodes to reduce the burden [23]. So, external or internal threats exist that can endanger the integrity of training data and lead to the wrong training results. Therefore, it is crucial to protect the training data’s integrity. The proposed scheme in this paper is suitable for overcoming this challenge. The proposed scheme can increase efficiency and reduce the costs needed to perform the data integrity verification scheme by enabling multi-verifiers and batch verification. Furthermore, this scheme can also resist malicious CSPs, verifiers, and COs that want to manipulate the verification results.

Big data: This proposed scheme is also a suitable solution in the case of a big data environment besides machine learning, such as medical, healthcare, and IoT data. In the medical or healthcare system, there will be vast quantities of patient information that needs to be stored over cloud computing systems [2], while, in the IoT environment, there is already growing data collection from various devices such as smart devices, household electronics, or vehicle monitoring equipment [22]. These kinds of data will also be stored in a multi-cloud environment, so the data integrity will need to be checked to ensure their trustworthiness. Therefore, with the property requirements and analyses presented in previous sections, this proposed scheme is relevant to solve these challenges.

## 8. Conclusions

This paper proposes a distributed data integrity verification in multi-cloud environments to increase the sampling rate in the probabilistic verification process without increasing the costs for the verifier. This paper presented a security analysis that proves the equation’s correctness and the proposed protocol’s unforgeability. Furthermore, this paper conducted several experiments to evaluate the performance of the proposed protocol. It shows that the distributed verification tasks with multi-verifiers can reduce time consumption compared to the single verifier. The numerical analysis also shows that the computation and communication costs are reduced in the case of multi-verifiers because the burden is distributed to multi-verifiers rather than only one verifier. Furthermore, by using batch verification, the costs for the CO are also lowered. The estimation of gas usage in smart contracts shows that the gas used between one verifier and multi-verifiers is the same. Lastly, the comparative studies also show that the proposed protocol can fulfill the important property requirements for data verification.

However, the limitation of the proposed scheme is that it does not support data dynamic property, which is also an important point when users store data in the cloud server. In cloud computing, users usually not only store data in the CSP. They may modify the data by updating, inserting, or deleting them. Therefore, this paper considers providing data dynamic property to the proposed data integrity verification scheme as part of future work.

## Figures and Tables

**Table 1:** Development environment of the user, CO, CSP, and verifier.

Parameter	User, CO, CSP, Verifier
CPU	Intel Core i5-7200U @2.50 GHz
Memory	4 GB
OS	Ubuntu 16.04 Xenial
Go	v.1.19.3
Library	PBC Go Wrapper, crypto-ecdsa

**Table 2:** Computation costs of each actor.

Actor	Computation Cost
User	(n×(Inv+Add+Mul+Hash))+2SIGN+VER
CSP	(c×(2Mul+Add+Hash))+SIGN+2VER
Verifier	((c×Mul)+(c×P))+2SIGN+2VER
CO	((t×Add)+P)+3SIGN+3VER

*c* = *n*/*a*, *Inv* = inverse, *Add* = addition, *Mul* = multiplication, *P* = bilinear pairing, *SIGN* = digital signature, and *VER* = verification of digital signature.

**Table 3:** Average computation time of each operation. AT = Average Time.

Operations	AT (ms)	Library
Inv	0.001354	PBC Go Wrapper
Mul	1.464053	PBC Go Wrapper
Add	0.000185	PBC Go Wrapper
Hash	0.000794	PBC Go Wrapper
*P*	1.125117	PBC Go Wrapper
SIGN	0.046341	Crypto-ecdsa
VER	0.101769	Crypto-ecdsa

**Table 4:** Communication costs of each actor.

User	Communication Cost
Send F+SIGN	(n×4000)+128
Send verification request + SIGN	6+128
**CSP**	**Communication Cost**
Send proof δ+SIGN	90+128
**Verifier**	**Communication Cost**
Send chal+SIGN	(c×47)+128
Send proof δ,ω+SIGN	180+128
**CO**	**Communication Cost**
Send task+SIGN	(a×(c+200))+128
Send result+SIGN	5+128

The measurement unit is in bytes.

**Table 5:** Estimation of gas usage for each function in the smart contract. * Data calculated from ETH Gas Station [28] on 31 October 2022. The average gas price at that time is 14 Gwei.

Number	Function	Estimation Gas Usage	Estimation Fee (USD) *	Actor
1	AddNewUser	50,733	1.15	User
2	AddNewVerifier	50,733	1.15	Verifier
3	AddNewCO	50,733	1.15	CO
4	AddNewUserData	107,399	2.42	CO
5	AddTask	171,257	3.86	CO
6	AssignVerifier	179,948	4.09	CO
7	SetTaskState	32,141	0.72	CO

**Table 6:** Properties requirements comparison.

Ref	Batch Verification	Multi- Cloud	Reliable CO	Reliable Verifiers	Blockchain- Based	Distributed Verification
[7]	×	×	×	*√*	*√*	×
[5]	×	×	×	*√*	*√*	×
[6]	*√*	*√*	*√*	*√*	*√*	×
[17]	×	×	×	×	×	×
[18]	×	*√*	*√*	*√*	*√*	×
[19]	*√*	×	×	*√*	*√*	×
[20]	×	×	×	×	×	×
[21]	×	×	×	×	×	×
[22]	×	×	×	*√*	*√*	×
[23]	×	×	×	×	×	×
Ours	*√*	*√*	*√*	*√*	*√*	*√*

## Data Availability

Not applicable.

## Nomenclature

$G_{1}$	GDH group
$G_{2}$	Multiplicative cyclic group
*P*	Generator of group $G_{1}$
*x*	ZSS Signature secret key
Ppub	ZSS Signature public key
*F*	Stored user’s file
*n*	Numbers of data blocks
$m_{i}$	User’s data index of *i*
Sign	Generated ZSS Signature
*S*	Set of Sign
$SIGN_{SK}$	Digital signature using the secret key of the actor
*a*	Number of verifiers
*c*	Number of challenged data blocks
*I*	Set of challenged data blocks
δ	Proof generated by CSP
ω	Proof generated by verifier
*k*	Index of generated proof δ or ω
*K*	Set of *k*
*t*	Number of proofs
*v*	Number of blocks stored in CSP
