# Correction: Witanto et al. Distributed Data Integrity Verification Scheme in Multi-Cloud Environment. *Sensors* 2023, *23*, 1623

**DOI:** 10.3390/s23125566

**Published:** 2023-06-14

**Authors:** Elizabeth Nathania Witanto, Brian Stanley, Sang-Gon Lee

**Affiliations:** College of Software Convergence, Dongseo University, Busan 47011, Republic of Korea

The authors make the following corrections to the published paper [1].

The errors appeared in the text because of the explanation in the following.

The original Equation (7):

(7) $$\begin{array}{cc} e(\delta_{k},\omega_{k})\; & =e(\sum_{i\in I}r_{i}\left(H\left(m_{i}\right)P+P_{pub}\right),\sum_{i\in I}r_{i}Sign_{i})\\ \; & =e(\sum_{i\in I}r_{i}\left(H\left(m_{i}\right)+x\right)P,\sum_{i\in I}r_{i}{\left(H\left(m_{i}\right)+x\right)}^{-1}P)\\ \; & =e{\left(P,P\right)}^{\sum_{i\in I}r_{i}\left(H\left(m_{i}\right)+x\right)\cdot \sum_{i\in I}r_{i}{\left(H\left(m_{i}\right)+x\right)}^{-1}}\\ \; & =e(P,P) \end{array}$$

is complex; thus, the authors made a mistake. The authors misunderstood that the exponential on the 3rd line could be canceled. Then, the authors discovered that the 3rd line of Equation (7) could not be canceled because the authors used a summation. Therefore, the authors have changed it to multiplication instead.

However, this mistake affects Equations (3), (4), (6), (8), and (9), which are strongly related to Equation (7). It also affects explanations in other paragraphs related to computational cost and experiments using our equations. The details of the changes are written in the following.

**Changes to Section 5. Proposed Scheme**

In Equation (3),

(3) $$\delta_{k}=\sum_{i\in I}r_{i}\left(H\left(m_{i}\right)P+P_{pub}\right)$$

should be changed to

(3) $$\delta_{k}=\prod_{i\in I}r_{i}\left(H\left(m_{i}\right)P+P_{pub}\right)$$

In Equation (4),

(4) $$\omega_{k}=\sum_{i\in I}r_{i}Sign_{i}$$

should be changed to

(4) $$\omega_{k}=\prod_{i\in I}Sign_{i}{r_{i}}^{-1}$$

In Equation (6),

(6) $$e\left(\sum_{k\in K}\delta_{k},\sum_{k\in K}\omega_{k}\right)=e\left(P,P\right)$$

should be changed to

(6) $$e\left(\prod_{k\in K}\delta_{k},\prod_{k\in K}\omega_{k}\right)=e\left(P,P\right)$$

**Changes to Section 6.1. Correctness**

In Equation (7),

(7) $$\begin{array}{cc} e(\delta_{k},\omega_{k})\; & =e(\sum_{i\in I}r_{i}\left(H\left(m_{i}\right)P+P_{pub}\right),\sum_{i\in I}r_{i}Sign_{i})\\ \; & =e(\sum_{i\in I}r_{i}\left(H\left(m_{i}\right)+x\right)P,\sum_{i\in I}r_{i}{\left(H\left(m_{i}\right)+x\right)}^{-1}P)\\ \; & =e{\left(P,P\right)}^{\sum_{i\in I}r_{i}\left(H\left(m_{i}\right)+x\right)\cdot \sum_{i\in I}r_{i}{\left(H\left(m_{i}\right)+x\right)}^{-1}}\\ \; & =e(P,P) \end{array}$$

should be changed to

(7) $$\begin{array}{cc} e(\delta_{k},\omega_{k})\; & =e(\prod_{i\in I}r_{i}\left(H\left(m_{i}\right)P+P_{pub}\right),\prod_{i\in I}Sign_{i}{r_{i}}^{-1})\\ \; & =e(\prod_{i\in I}r_{i}\left(H\left(m_{i}\right)+x\right)P,\prod_{i\in I}{\left(r_{i}\left(H\left(m_{i}\right)+x\right)\right)}^{-1}P)\\ \; & =e{\left(P,P\right)}^{\prod_{i\in I}r_{i}\left(H\left(m_{i}\right)+x\right)\cdot \prod_{i\in I}{\left(r_{i}\left(H\left(m_{i}\right)+x\right)\right)}^{-1}}\\ \; & =e(P,P) \end{array}$$

In Equation (8),

(8) $$\begin{array}{cc} e(\sum_{k\in K}\delta_{k},\sum_{k\in K}\omega_{k})\; & =e(\sum_{k\in K}\sum_{i\in I}r_{ki}\left(H\left(m_{ki}\right)P+P_{pub}\right),\sum_{k\in K}\sum_{i\in I}r_{ki}Sign_{ki})\\ \; & =e(\sum_{k\in K}\sum_{i\in I}r_{ki}\left(H\left(m_{ki}\right)+x\right)P,\sum_{k\in K}\sum_{i\in I}r_{ki}{\left(H\left(m_{ki}\right)+x\right)}^{-1}P)\\ \; & =e{\left(P,P\right)}^{\sum_{k\in K}\sum_{i\in I}r_{ki}\left(H\left(m_{ki}\right)+x\right)\cdot \sum_{k\in K}\sum_{i\in I}r_{ki}{\left(H\left(m_{ki}\right)+x\right)}^{-1}}\\ \; & =e(P,P) \end{array}$$

should be changed to

(8) $$\begin{array}{cc} e(\prod_{k\in K}\delta_{k},\prod_{k\in K}\omega_{k})\; & =e(\prod_{k\in K}\prod_{i\in I}r_{ki}\left(H\left(m_{ki}\right)P+P_{pub}\right),\prod_{k\in K}\prod_{i\in I}Sign_{ki}{r_{ki}}^{-1})\\ \; & =e(\prod_{k\in K}\prod_{i\in I}r_{ki}\left(H\left(m_{ki}\right)+x\right)P,\prod_{k\in K}\prod_{i\in I}{\left(r_{ki}\left(H\left(m_{ki}\right)+x\right)\right)}^{-1}P)\\ \; & =e{\left(P,P\right)}^{\prod_{k\in K}\prod_{i\in I}r_{ki}\left(H\left(m_{ki}\right)+x\right)\cdot \prod_{k\in K}\prod_{i\in I}{\left(r_{ki}\left(H\left(m_{ki}\right)+x\right)\right)}^{-1}}\\ \; & =e(P,P) \end{array}$$

**Changes to Section 6.2. Unforgeability**

In Equation (9),

(9) $$\delta^{\prime }=\sum_{i\in I,i\neq j}r_{i}H\left(m_{i}\right)P+r_{j}H\left(m_{b}\right)P$$

should be changed to

(9) $$\delta^{\prime }=\prod_{i\in I,i\neq j}r_{i}H\left(m_{i}\right)P+r_{j}H\left(m_{b}\right)P$$

**Changes to Section 7.1. Computation Cost**

In paragraph 1, the sentence "The cost of the CSP is (c×(2Mul+Add+Hash))+SIGN+2VER with the bracket showing the cost for generating proof δ, while the cost of the verifier is ((c×Mul)+(c×P))+2SIGN+2VER with the bracket showing the cost for generating proof ω and bilinear pairing of proofs δ,ω in the verification process. The last is the cost of CO, ((t×Add)+P)+3SIGN+3VER with the bracket showing the cost for the batch verification process." should be changed to:

“The cost of the CSP is (c×(3Mul+Add+Hash))+SIGN+2VER with the bracket showing the cost for generating proof δ, while the cost of the verifier is ((c×Mul×Inv)+(c×P))+2SIGN+2VER with the bracket showing the cost for generating proof ω and bilinear pairing of proofs δ,ω in the verification process. The last is the cost of CO, ((t×Mul)+P)+3SIGN+3VER, with the bracket showing the cost for the batch verification process.”

Table 2 “Computation costs of each actor” was shown in the text as:

**Table 2:** Computation costs of each actor.

Actor	Computation Cost
User	(n×(Inv+Add+Mul+Hash))+2SIGN+VER
CSP	(c×(2Mul+Add+Hash))+SIGN+2VER
Verifier	((c×Mul)+(c×P))+2SIGN+2VER
CO	((t×Add)+P)+3SIGN+3VER

c=n/a, Inv = inverse, Add = addition, Mul = multiplication, *P* = bilinear pairing, SIGN = digital signature, VER = verification of digital signature.

It should be changed to

**Table 2:** Computation costs of each actor.

Actor	Computation Cost
User	(n×(Inv+Add+Mul+Hash))+2SIGN+VER
CSP	(c×(3Mul+Add+Hash))+SIGN+2VER
Verifier	((c×Mul×Inv)+(c×P))+2SIGN+2VER
CO	((t×Mul)+P)+3SIGN+3VER

c=n/a, Inv = inverse, Add = addition, Mul = multiplication, *P* = bilinear pairing, SIGN = digital signature, and VER = verification of digital signature.

**Changes to Section 7.3. Experiment Results**

In paragraph 1, the sentence “CSP reaches time 5.6 s for generating proof δ of 2000 data blocks and the user 2.6 s for generating ZSS signature of the same amount of data blocks. CSP needs a longer time because as shown in Equation (3), it needs two multiplication operations. Different from the user that only needs one multiplication operation in Equation (2).” should be changed to:

“The CSP reaches time 5.3 s for generating proof δ of 2000 data blocks and the user 2.6 s for generating the ZSS signature of the same amount of data blocks. The CSP needs a longer time because, as shown in Equation (3), it needs three multiplication operations. Different from the user that only needs one multiplication and one inverse operation in Equation (2).”

In the original article, due to the correction to Equation (3), a change is required to Figure 5. The corrected Figure 5 appears below.

In paragraph 3, the sentence “It needs 10 s to verify 2000 data blocks. However, the case of multi-verifiers (5, 10, 15, and 20 verifiers) reduces the time consumption significantly with results of 1.9 s, 1 s, 0.6 s, and 0.5 s, respectively, for the same amount of data blocks.” should be changed to:

“It needs 7.3 s to verify 2000 data blocks. However, the case of multi-verifiers (5, 10, 15, and 20 verifiers) significantly reduces the time consumption with results of 1.5 s, 0.7 s, 0.5 s, and 0.4 s, respectively, for the same amount of data blocks.”

In the original article, due to the correction to Equation (4), a change is required to Figure 6. The corrected Figure 6 appears below.

The authors apologize for any inconvenience caused and state that the scientific conclusions are unaffected. The original article has been updated.

## Figures and Tables

**Figure 5:**
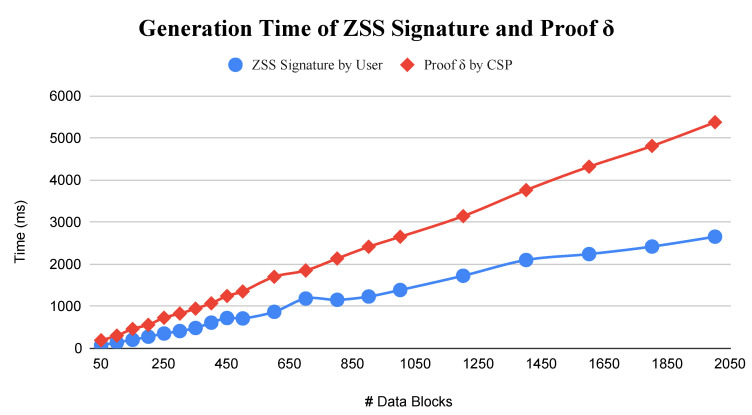
Generation time of Signature and Proof Delta.

**Figure 6:**
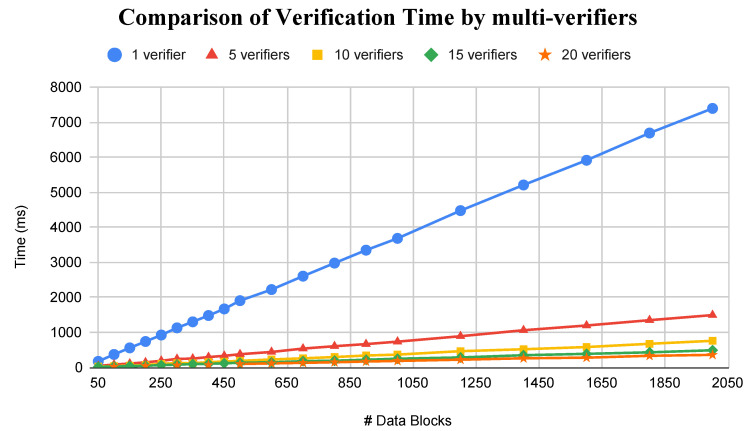
Comparison of verification time using multi-verifiers.
